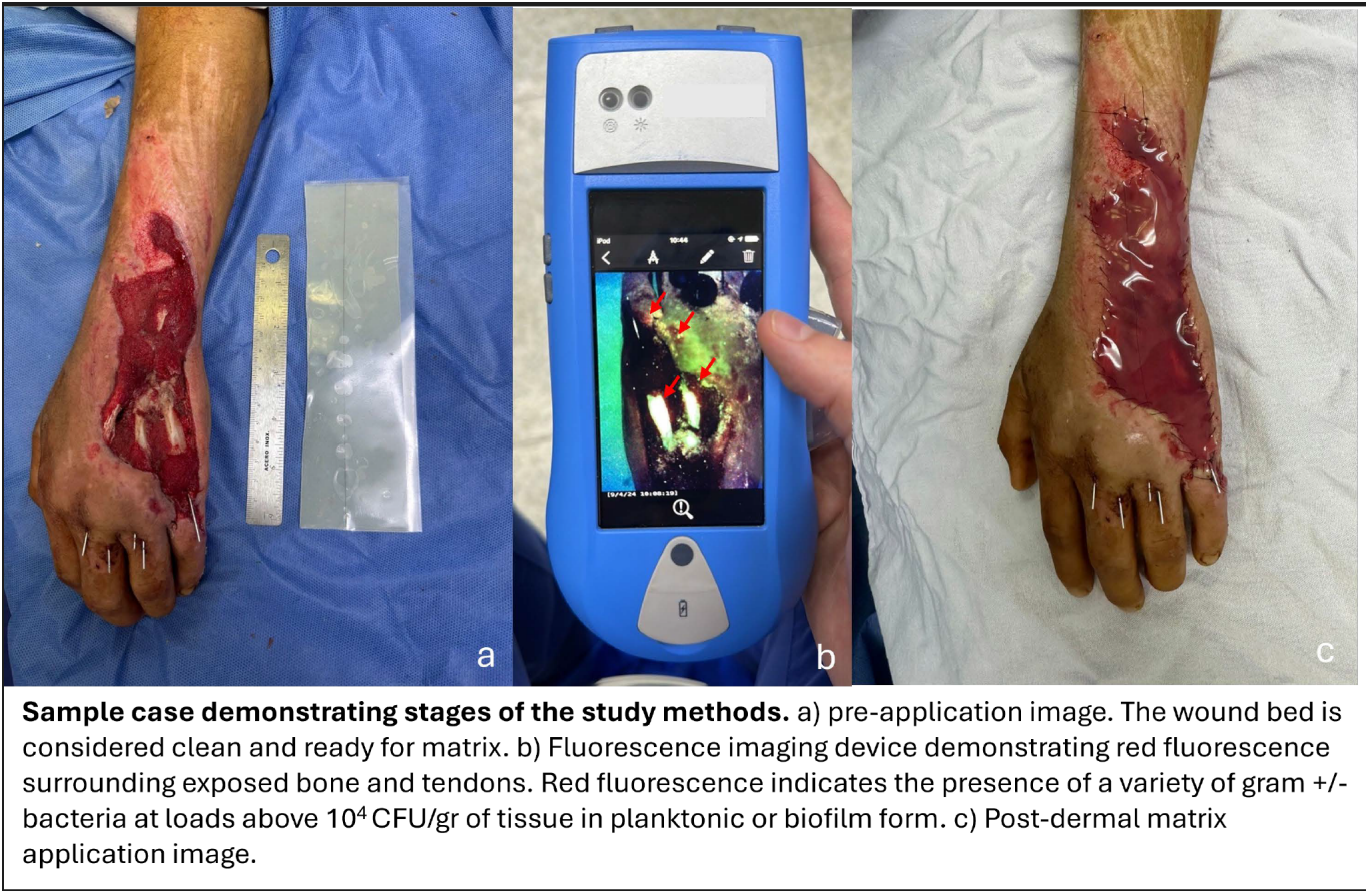# 562 Bacterial Fluorescence Signals Are Associated with Dermal Regeneration Template Infections in Burn Patients

**DOI:** 10.1093/jbcr/iraf019.191

**Published:** 2025-04-01

**Authors:** Erik Hanson Viana

**Affiliations:** Hospital Angeles

## Abstract

**Introduction:**

Dermal regeneration templates (DRT) can prepare wound beds for split-thickness skin grafting (STSG), however early signs of bacteria/biofilm presence may be covert and can be obscured by the silicone layer complicating wound assessment and leading to undetected infections and matrix failure. Non-invasive fluorescence (FL) imaging detects high bacterial loads and biofilm in real-time, and its signals have shown a strong regional correlation with STSG outcomes in burn patients (1). Here, we assessed if this correlation is also evident when considering bacterial infection outcomes following DRT placement.

**Methods:**

Single-center prospective observational study including 20 adult burn patients undergoing DRT placement over areas with exposed bone/tendon prior STSG. All confirmed clinically and microbiologically negative for previously treated infection. FL imaging found bacteria/biofilms, when positive indicating >10⁴ CFU/g loads. The surgical team was blinded to FL results, and DRT infection/integration was recorded 2-4 weeks later. A 2x2 contingency table evaluated the predictive power of FL signals for infection, and Fisher’s exact test assessed the association between FL results and infection or seroma presence.

**Results:**

Of the 20 patients enrolled in the study, bacterial FL seen in and around the graft bed was detected in 40% (8 patients) and absent in 60% (12 patients). Among those with positive FL, 4 out of 8 patients developed an infection and seroma, while 3 presented with seroma approximately 3 weeks post-matrix application. The Sensitivity of FL was 100% in relation to infection/seroma events. The Specificity was 92%, PPV 88% and NPV 100%. When re-calculating using infection only as the event of interest (excluding seroma), only Specificity (75%) and the PPV (50%) decreased while other measures remained unchanged. Of note, 3 out of the 4 infections were positive for Pseudomonas on microbiology. The association of instances of infection/seroma and FL was statistically significant.

**Conclusions:**

FL signals arising from high bacterial loads were more common in burn patients who developed post-operative infections of DRTs, suggesting a link between the presence of these signals before application and post-operative infection/seroma risk. This aligns with previous studies involving FL-imaging and can if used as guidance can help prepare the wound bed and time DRT application in a way that improves outcomes. The series is being expanded at present to strengthen the validity of the findings herein.

**Applicability of Research to Practice:**

Real-time fluorescence signals indicating bacteria/biofilm may better inform graft readiness and reduce rates of post-operative infections.

**Funding for the Study:**

The FL-imaging device was loaned at no cost by the local distributor for independent research purposes.